# Determinants of influenza non-vaccination among Canadian children: insights from a nationwide survey

**DOI:** 10.3389/fpubh.2024.1400782

**Published:** 2024-06-05

**Authors:** Abdallah Alami, Sailly Dave, Caren Uhlik, Marwa Ebrahim, Daniel Krewski, Julie Laroche

**Affiliations:** ^1^Vaccine Coverage and Effectiveness Surveillance Division, Infectious Diseases and Vaccination Programs Branch, Public Health Agency of Canada, Ottawa, ON, Canada; ^2^School of Epidemiology and Public Health, Faculty of Medicine, University of Ottawa, Ottawa, ON, Canada

**Keywords:** influenza, survey, vaccine hesitancy, public health, immunization, COVID-19, Canada

## Abstract

**Background:**

To identify determinants influencing Canadian parents’ decision not to vaccinate their children aged 6 months to 17 years against seasonal influenza.

**Methods:**

Data from the 2022 Childhood COVID-19 Immunization Coverage Survey, a national survey of approximately 10,500 Canadian parents/guardians and their children, was analyzed. The survey examined influenza vaccine coverage, parental perspectives on vaccines, reasons for hesitancy, and factors influencing immunization. Socio-demographic characteristics, including ethnicity, household income, working sector, educational attainment, and prevalence of chronic medical conditions among children were considered. Historical vaccine uptake and the impact of the COVID-19 pandemic on immunization decisions were also reviewed. Key determinants of non-vaccination in the 2021–2022 influenza season were analyzed using multivariable logistic regression, with a statistical significance level set at *p*-value <0·05.

**Results:**

70% of children aged 6 months to 17 years did not receive the seasonal influenza vaccine. Key predictors for non-vaccination included: residing in rural settings (aOR 1·35, 95% CI 1·13–1·60), parental education attainment of less than high school (aOR 2·48, 95% CI 1·24–4·97), and the absence of chronic medical conditions in children (aOR 1.60, 95% CI 1.34-1.91)· Other strong predictors included lower household income; deterrence due to the COVID-19 pandemic; and parental hesitancy stemming from concerns about the vaccine’s safety, effectiveness, and by beliefs that their child was not at risk of contracting the influenza or severe consequences from the infection.

**Conclusion:**

This research underscores pivotal determinants of parental decisions not to vaccinate their children against seasonal influenza and sheds light on the impact of the COVID-19 pandemic. The results highlight the importance of addressing safety concerns and providing clear information to alleviate hesitancy.

## Introduction

Childhood vaccination is fundamental in maintaining the health of both individuals and the broader community, especially when it comes to the seasonal influenza. This respiratory illness, though common, can lead to severe health complications, especially in young children and people over 65 years of age, as well as in those with underlying chronic health conditions (1, 2).

Children are particularly vulnerable to the seasonal influenza, with data from Canada showing they are disproportionately affected (3). Complications from influenza in children can include pneumonia, dehydration, and worsening of long-term medical problems such as heart disease or asthma, sinus problems, and ear infections (4). In rare cases, influenza complications can lead to death (4). According to the Canadian Immunization Monitoring Program Active (IMPACT) surveillance network, between 2004 and 2005 and 2012–2013 (excluding the 2009–2010 pandemic season) pediatric seasonal influenza was confirmed in 15·5% to 58·3% of hospital admissions in children 16 years of age and younger (5, 6). In the wake of the COVID-19 pandemic, the 2022–2023 seasonal influenza season in Canada marked a significant shift, resembling pre-pandemic seasonal influenza activity but with notable impact on pediatric population (7). According to the National Influenza Annual Report, Canada, 2022–2023, which draws on FluWatch data (a long-standing national surveillance system monitoring the spread of influenza and influenza-like illness in Canada), nearly half (45%; n = 6,194/13,729) of the reported influenza A detections occurred in the pediatric population (younger than 19 years) (7). Furthermore, when hospitalizations are broken down by type, the pediatric population accounted for 49% of hospitalizations associated with influenza B, compared to 22% for influenza A (7). Additionally, during this period, weekly pediatric influenza-associated hospital admissions persistently exceeded historical peak levels, with children aged 0–4 years being the most affected group, experiencing the highest cumulative hospitalization rate at 131 per 100,000 population (7).

Given these findings, and to mitigate the potential complications of seasonal influenza infection in children, the National Advisory Committee on Immunization (NACI) continues to recommend that the seasonal influenza vaccine should be offered annually to anyone 6 months of age and older who does not have a contraindication to the vaccine (5). Despite the availability of an effective vaccine, many parents are choosing not to vaccinate their children against the influenza (8). Gaining insights into the reasons behind these decisions is critical not only to boost vaccination rates, but also to ensure the broader community remains protected. With vaccine hesitancy frequently expressed during the recent COVID-19 pandemic, monitoring and understanding parental attitudes toward vaccination is critical to increasing seasonal influenza vaccination uptake. Parental perceptions can provide insight into expected vaccine uptake and can help shape public educational and awareness campaigns. This is particularly important given the high degree of uncertainty in estimates of seasonal influenza vaccine coverage in children. This study aims to explore factors associated with non-vaccination against the seasonal influenza in Canadian parents of children aged 6 months to 17 years old. The results of this study can provide valuable insights into parental attitudes and beliefs about influenza vaccination and inform the development of targeted interventions aimed at increasing seasonal influenza vaccination rates and protecting public health.

## Methods

### Data source

This study utilizes data from the 2022 Childhood COVID-19 Immunization Coverage Survey (CCICS) (9), an annual survey first implemented by the Public Health Agency of Canada in 2022. Data collection for this survey was conducted over a period extending from April 20 to July 21, 2022. The survey constitutes a nationally representative dataset for Canadian parents or guardians with children in specific age groups (0–4, 5–11, and 12–17 years). CCICS encompasses all Canadian provinces and territories and ensures a balanced representation of males and females. As a surveillance tool, CCICS provides both national and provincial/territorial-level estimates of several key factors, including seasonal influenza vaccine coverage among eligible children; knowledge, attitudes, and beliefs (KAB) of respondents toward vaccinations; and barriers and facilitators to immunization. The survey also provides information about the impact of the COVID-19 pandemic on vaccination decisions, as well as vaccination history and uptake among children and their parents or guardians. Socio-demographic data including household income, working sector, education, and citizenship status in Canada are also collected in the survey.

### Study design

This cross-sectional study employed a probability-based sampling strategy, where the CCICS aimed for a sample size of 10,500 Canadian parents or guardians 18 years of age or older. To achieve a nationally representative sample, respondents were recruited from a general population sample by random digit dialing (RDD) (10), across all provinces and territories. The sampling framework allows for extrapolation to the broader Canadian population. To strengthen statistical power and ensure national representativeness, quotas were set for key sub-populations.^1^ Survey sampling weights were applied to mirror the demographic composition of the Canadian population of children, based on child’s sex, child’s age group and province or territory of residence (based on the most recent data from the 2021 Statistics Canada census). Bootstraps were generated and applied to estimate variance. Overall, 100·3% of the target sample size was successfully achieved, ensuring that the study had adequate power.

### Data collection

Data was captured using a multimodal approach, administered either online or through computer assisted telephone interviewing (CATI). CATI was specifically done in hard-to-reach populations to increase response rates (targeting parents in Atlantic and Northern provinces/territories who are often more difficult to reach online). This flexible approach facilitated comprehensive data collection across diverse demographic groups.

### Inclusion and exclusion criteria

In alignment with Health Canada’s seasonal influenza vaccination authorization starting at 6 months of age, this analysis purposely excludes data on children younger than 6 months. Results are therefore based on survey responses from parents or guardians of children aged 6 months to 17 years across all Canadian provinces and territories. Missing data were not expected to pose a significant issue. A threshold for data removal was established: any variable exhibiting more than 20% missing data was excluded from the regression analysis. To assess the randomness of missing values in the dataset, we generated graphical summaries visualizing the patterns of missingness across variables: this visualization was performed for variables with more than 5% of missing data, allowing for an evaluation of whether the missing values demonstrated any discernable trends or patterns (Supplementary material, Supplementary Figure S1).

### Statistical and data analysis

R Statistical Software (version 4·1·3; R Foundation for Statistical Computing, Vienna, Austria) was used for data analysis, including data filtering, analysis and wrangling, and both descriptive and inferential statistical analyses (11). Initial data exploration involved summarizing categorical dependent and independent variables using descriptive statistics. Unweighted and weighted frequencies and proportions were calculated, stratified by seasonal influenza vaccination status. Likert plots were used to summarize parental opinions on key KAB questions about vaccines: this graphical representation displayed the range of parental responses, from ‘Strongly Agree’ to ‘Strongly Disagree,’ for various belief statements about vaccine safety and effectiveness.

In line with our research objectives, we analyzed and incorporated parental hesitancy, along with its underlying reasons, to better understand determinants of children’s non-vaccination against seasonal influenza. In the survey, parents were asked about their hesitancy to vaccinate their child against the influenza during the 2021–2022 season. Those who indicated hesitancy were prompted to select their specific reason(s). Given that respondents could choose multiple reasons, we categorized these into three broad categories for analytical purposes:

1. concerns about vaccine effectiveness, safety, and perceived risk which encompassed doubts about the influenza vaccine’s efficacy, children’s susceptibility to flu, potential vaccine side effects, concerns regarding combined influenza and COVID vaccinations, and past adverse vaccine events;
2. barriers related to access and information availability, including hesitancy due to challenges in discussing the influenza vaccine with healthcare experts, sourcing trustworthy information, and unfavorable encounters with medical practitioners; and
3. personal beliefs and other influences, such as religious or philosophical stances and fears of racism or discrimination.

From these categories, we developed a five-level classification system for analysis:

- “Not Hesitant,”
- “Hesitant: Effectiveness/Safety/Perceived Risk” (derived from the first category),
- “Hesitant: Access/Information” (from the second category),
- “Hesitant: Personal/Other Influences” (reflecting the third category),
- “Hesitant: Multiple Reasons” (for those indicating reasons across multiple categories).

This structured approach not only enriched our understanding of the multifaceted influences on vaccination hesitancy, but also was instrumental for our regression models. The five-level classification system facilitated a comprehensive exploration of the relationship between influenza vaccine hesitancy, vaccine uptake, and the specific parental reasons behind hesitancy.

A multivariable logistic regression model was employed to explore factors associated with non-vaccination of children aged 6 months to 17 years against the seasonal influenza. To account for the complexities of our survey design, we estimated standard errors, coefficients of variation, and confidence intervals using the bootstrap technique (12). As a starting point, univariate logistic regression analyses were fit to the survey data for each predictor to derive unadjusted odds ratios (ORs), with confidence interval (CI) set at 95%. Variables not achieving statistical significance at this stage were not immediately excluded; rather, as their effect could become apparent in a multivariable context after controlling for other variables, they were earmarked for potential removal in later stages.

To address multicollinearity among categorical predictors, we employed Cramer’s V, guided by thresholds established by Lee et al. (13). Predictors exceeding a Cramer’s V value of 0·4 were flagged for removal to mitigate the effects of a possible multicollinearity. We adopted a ‘best fit’ strategy for construction multivariable models. This entailed initially incorporating all relevant predictors, including those that showed statistically insignificant effects in the univariate analysis but were deemed practically significant. A stepwise backward elimination process was then conducted, guided by the Akaike Information Criterion (AIC), *p*-values, and adjustments for multiple comparisons. The final model was selected based on a combination of statistical significance, minimized AIC values and domain expertise. Adjusted odds ratios (aOR) were used to evaluate relationships between predictors and the outcome of non-vaccination among children. A likelihood ratio test was used to validate the goodness of fit of the final model (14).

An odds ratio plot was generated using ggplot2 and Finalfit packages in R (15) to visualize predictors and their respective aORs in the final model: this plot included 95% CI on the aORs to gage the impact of each variable on the likelihood of non-vaccination in children.

## Results

### Sociodemographic characteristics of the survey population

A total of 10,536 individuals participated in the survey, achieving an overall response rate of 26·1%.

Of these, 10,236 respondents were included in the analysis, as responses from parents or guardians of children under 6 months of age were not included. Table 1 presents the sociodemographic characteristics of the survey population, stratified by vaccination status. This table provides a comprehensive overview of the sample population across different variables, including province/territory, age of child and responding parent, sex, urban/rural setting, ethnicity, and household income.

**Table 1:** Sociodemographic characteristics of survey respondents, stratified by child seasonal influenza vaccination status.

	Overall	Vaccinated children	Unvaccinated children
**Characteristic**	**N (%)** ^1^	**N (%)** ^1^	**N (%)** ^1^
**Sample size**	10,236 (100·0)	3,230 (30·0)	7,006 (70·0)
**Province/Territory**			
Alberta (AB)	1,240 (13·5)	463 (17·3)	777 (11·8)
British Columbia (BC)	1,342 (12·1)	444 (13·6)	898 (11·5)
Manitoba (MB)	399 (4·3)	180 (6·6)	219 (3·3)
New Brunswick (NB)	382 (1·9)	139 (2·2)	243 (1·7)
Newfoundland and Labrador (NL)	389 (1·2)	168 (1·7)	221 (0·9)
Nova Scotia (NS)	408 (2·3)	159 (3·0)	249 (2·0)
Ontario (ON)	3,019 (37·9)	932 (39·3)	2,087 (37·4)
Prince Edward Island (PE)	386 (0·4)	165 (0·6)	221 (0·3)
Quebec (QC)	1,945 (22·2)	251 (9·5)	1,694 (27·6)
Saskatchewan (SK)	404 (3·8)	175 (5·5)	229 (3·1)
Territories	322 (0·4)	154 (0·7)	168 (0·3)
**Child age**			
6 months – 4 years	2,551 (25·1)	1,047 (33·4)	1,504 (21·5)
5–11 years	3,724 (40·4)	1,166 (39·1)	2,558 (40·9)
12–17 years	3,961 (34·6)	1,017 (27·6)	2,944 (37·6)
**Age of responding parent**			
18–29	201 (1·7)	50 (0·9)	151 (2·0)
30–39	2,968 (30·0)	1,039 (33·0)	1,929 (28·7)
40–49	4,932 (49·2)	1,511 (47·9)	3,421 (49·8)
50+	2,025 (19·1)	612 (18·1)	1,413 (19·6)
**Child sex at birth**			
Male	5,306 (51·0)	1,656 (50·2)	3,650 (51·3)
Female	4,930 (49·0)	1,574 (49·8)	3,356 (48·7)
**Sex of responding parent**			
Male	3,990 (39·2)	1,209 (37·5)	2,781 (39·9)
Female	6,191 (60·8)	2,015 (62·5)	4,176 (60·1)
**Urban/rural setting**			
Urban	8,415 (86·1)	2,743 (89·7)	5,672 (84·5)
Rural	1,737 (13·9)	475 (10·3)	1,262 (15·5)
**Ethnicity of responding parent**			
Black	267 (2·9)	38 (1·3)	229 (3·6)
East/Southeast Asian	457 (4·4)	176 (5·5)	281 (3·9)
South Asian descent	304 (3·5)	94 (3·4)	210 (3·5)
Latin American	153 (1·7)	44 (1·4)	109 (1·8)
Middle Eastern and North African	222 (2·5)	54 (1·9)	168 (2·7)
Indigenous	158 (0·9)	52 (0·9)	106 (0·9)
White European descent	7,825 (79·1)	2,572 (80·8)	5,253 (78·3)
Other/Mixed parent ethnicity	476 (5·1)	138 (4·8)	338 (5·3)
**Education of responding parent**			
Less than high school	134 (1·1)	25 (0·4)	109 (1·4)
High school or equivalent	808 (7·3)	212 (6·1)	596 (7·9)
Postsecondary below Bachelor’s	3,247 (31·5)	766 (22·1)	2,481 (35·6)
Bachelor’s or above	5,926 (60·0)	2,209 (71·3)	3,717 (55·1)
**Status of residency in Canada**			
Canadian by birth	8,111 (78·5)	2,684 (82·1)	5,427 (76·9)
Canadian by naturalization	1,578 (17·0)	405 (14·1)	1,173 (18·3)
Permanent resident/landed immigrant	447 (4·1)	122 (3·5)	325 (4·4)
Refugee claimant/Asylum seekers	9 (0·1)	4 (0·1)	5 (0·1)
Temporary resident	36 (0·2)	9 (0·2)	27 (0·3)
**Working sector of responding parent**			
High-risk sector^2^	3,970 (39·4)	1,326 (40·4)	2,644 (38·9)
Not high-risk sector	5,969 (60·6)	1,859 (59·6)	4,110 (61·1)
**Total household income ($)**			
Under 40,000	650 (6·7)	136 (4·5)	514 (7·6)
40,000-59,999	721 (7·3)	153 (4·5)	568 (8·5)
60,000-79,999	870 (8·9)	222 (6·6)	648 (9·9)
80,000-99,999	1,152 (11·9)	324 (10·1)	828 (12·7)
100,000-149,999	2,502 (27·0)	776 (25·5)	1,726 (27·7)
150,000 and above	3,522 (38·2)	1,414 (48·8)	2,108 (33·5)
**Prevalence of chronic conditions among children**			
With chronic condition^3^	1,285 (12·4)	473 (14·7)	812 (11·4)
Without chronic condition	8,835 (87·6)	2,729 (85·3)	6,106 (88·6)
**Prevalence of disabilities among children**			
With disability^4^	675 (6·5)	197 (6·0)	478 (6·7)
Without disability	9,477 (93·5)	3,011 (94·0)	6,466 (93·3)

1 Percent weighted by: region, child’s age group, and child’s sex at birth.

2 High-Risk Working Sector: This category includes individuals currently employed or volunteering in sectors with elevated exposure risks. These sectors encompass healthcare, laboratory services, childcare, educational institutions, occupations with animal exposure, emergency services, and other critical roles such as staff in correctional facilities, crew on ships or aircraft, military personnel, humanitarian relief workers, and providers of essential community services.

3 Chronic Medical Condition: This refers to health conditions as outlined in the Canadian Immunization Guide and include sickle cell anemia or thalassemia major, neurological or neurodevelopmental disorders, asthma and other chronic respiratory diseases, chronic conditions affecting the liver, heart, or kidneys, diabetes, obesity, Down Syndrome, immune suppression (due to chemotherapy, radiotherapy, steroid use, HIV, organ transplants), cancer, and other significant medical conditions.

4 Disability: This is defined as a person who has a long-term or recurring impairment (such as vision, hearing, mobility, flexibility, dexterity, pain, learning, developmental, memory or mental health-related) which limits their daily activities inside or outside the home (such as at school, work, or in the community in general).

Thirty percent (30·0%, *N* = 3,230) of children younger than 18 years were vaccinated against seasonal influenza. A majority of the responding parents/guardians were female (60·8%) and of White European descent (79·1%). Nearly two thirds of the parents (60·0%) had a bachelor’s degree or higher, with 39·4% reporting being employed in high-risk sectors (including health care or laboratory workers, those working in child care or schools, those exposed to animals or their materials, and emergency services workers). Children ranged in age from 6 months to 4 years (25·1%), 5 to 11 years (40·4%), and 12 and 17 years (34·6%). A small minority of children had chronic medical conditions (12·4%) and disabilities (6·5%).

### Pre-Pandemic and current seasonal influenza vaccination patterns among parents and children

Prior to the COVID-19 pandemic, 42·7% of parents indicated that they typically received an seasonal influenza vaccine either every influenza season or most seasons. However, for the 2021–2022 influenza season, two-thirds (66·9%) of parents opted out of receiving the vaccine, while only one-third (33·1%) reported getting vaccinated (Table 2). When asked about their child’s seasonal influenza vaccination status prior to the COVID-19 pandemic, 38·4% of parents indicated vaccinating their child every or most influenza seasons, 18·5% reported having their children vaccinated sometimes, and 43·1% reported never vaccinating their child against the flu. A great majority of parents (93·3%) reported that their child had received all recommended routine vaccinations, with a further 4·2% receiving partial vaccinations, and 2·4% remaining unvaccinated.

**Table 2:** Parental and child seasonal influenza vaccination history, future intentions, and reasons for vaccination.

Vaccination history and reasons for vaccination	N (%)^1^
**Responding parent frequency of receiving an influenza vaccine prior to COVID-19**	
Every influenza (flu) season	2,475 (23·2)
Most influenza (flu) seasons	2,006 (19·5)
Some influenza (flu) seasons (including once only)	2,818 (27·8)
Never	2,894 (29·5)
**Responding parent influenza vaccination during the 2021–2022 influenza season**	
Yes, vaccinated	3,928 (37·1)
No, did not get the vaccine	6,263 (62·9)
**Uptake of recommended routine child vaccination**	
Complete	8,786 (93·3)
Partial	384 (4·2)
None	219 (2·4)
**Child frequency of receiving a influenza vaccine prior to the COVID-19 pandemic**	
Every influenza (flu) season	2,330 (24·3)
Most influenza (flu) seasons	1,354 (14·1)
Some influenza (flu) seasons (including once only)	1,783 (18·5)
Never	3,831 (43·1)
**How likely is it that you will get your child vaccinated against the influenza (flu) in the next influenza (flu) season?**	
Definitely will	2,871 (26·7)
Probably will	2,455 (23·6)
Probably will not	2,264 (22·9)
Definitely will not	1,836 (18·9)
Do not know	780 (7·9)
**Reasons for child to receive a influenza (flu) vaccine**	
To protect themselves and/or household members from the influenza (flu)	2,688 (84·5)
Based on public health recommendations	1,507 (46·1)
To prevent the spread of the flu in my community	1,594 (49·2)
The influenza (flu) vaccine was recommended by a health care professional	928 (27·7)
The influenza (flu) vaccine is available and free	1,343 (40·5)
Increased concerns about flu because of the COVID-19 pandemic	914 (27·9)
My child receives it every year	1,578 (48·9)
Other	49 (1·6)

1 Percent weighted by: region, child’s age group, and child’s sex at birth.

2 Multiple response options could be selected by respondents.

Concerning the likelihood of having their child vaccinated against seasonal influenza in the upcoming season, approximately half (50·3%) of parents signaled strong intent (either ‘definitely will’ or ‘probably will’) to have their children vaccinated; 22·9% of parents expressed a moderate likelihood of avoiding vaccination, saying they ‘probably will not’, while 18·9% expressed strong views against vaccination, responding with ‘definitely will not’.

The most common motivation for parents vaccinating their child was self and household protection from seasonal influenza (85·0%), followed by desire to prevent the spread of seasonal influenza in the community (49·0%), and the fact that the child receives the vaccine annually (48·0%).^2^

### Parental attitudes, influences, and barriers to seasonal influenza vaccination

**Figure 1:**
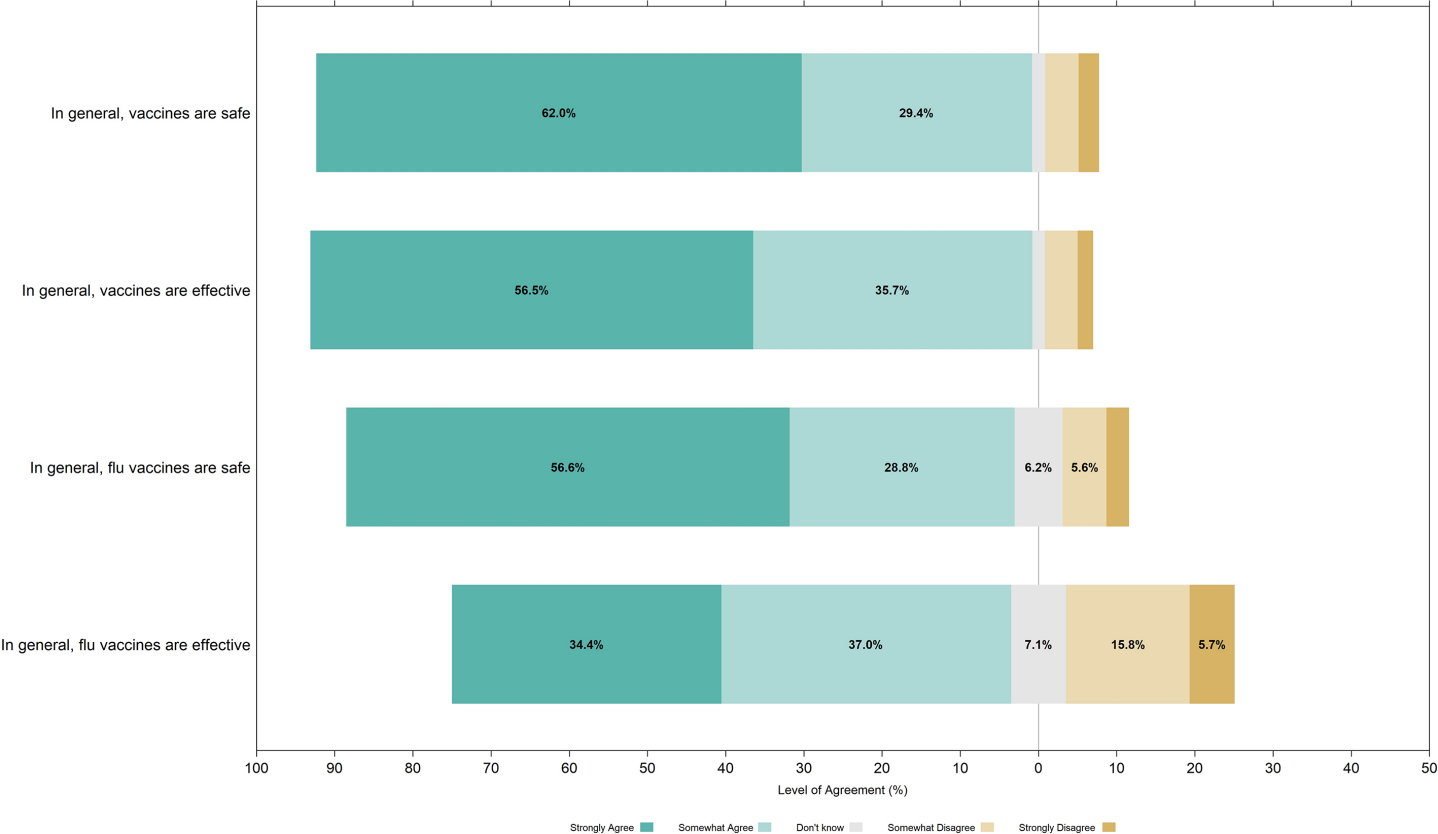
Parental attitudes toward vaccine safety and effectiveness: Likert plot distribution of responses on vaccine belief statements^1^. ^1^Percentages less than 5% are not numerically displayed for clarity.

A substantial majority of parents expressed favorable opinions regarding general vaccine safety and effectiveness. Specifically, 91·4% of parents either strongly or somewhat agreed that vaccines are generally safe, and 92·2% concurred on their effectiveness. Confidence levels in seasonal influenza vaccines varied somewhat: 85·4% of parents believed the influenza vaccine to be safe, and 71·4% attested to its effectiveness. A Likert plot displaying the range of responses on the various belief statements is provided in Figure 1, illustrating the distribution of opinions among survey respondents.

Approximately 24% of parents expressed hesitancy in vaccinating their child against seasonal influenza. The primary reason underlying this hesitancy was the belief that their child was not at risk of contracting influenza or developing severe symptoms, a view held by nearly half (47·2%) of these hesitant parents. Additional reservations were grounded in concerns about the influenza vaccine’s effectiveness (35%) and apprehensions regarding its safety or potential side effects (29·4%). These specific reasons for hesitancy are further detailed in Table 3. While the COVID-19 pandemic might have impacted seasonal influenza vaccination decisions for the 2021–2022 season, a notable majority (79·7%) of parents reported that the pandemic did not influence their choice. While the pandemic served as a motivator for 10·6% of parents, nearly an identical percentage found it to be a deterrent for vaccinating their child against the influenza. When examining the barriers that might have prevented parents from vaccinating children against seasonal influenza, the vast majority (81%) reported no obstacles to vaccination. Among the remaining parents who did face challenges, the barriers were diverse. Difficulty in securing time off from work or school was the most common obstacle, cited by 34% of these parents, followed by their child’s fear of needles (24%). Financial concerns were relatively rare, with only 3% citing the cost of the vaccine as a barrier. Other obstacles included limited access to transportation in remote areas (2%), language barriers (0·5%), and concerns about racism or discrimination (0·9%)·.

**Table 3:** Knowledge, attitudinal and behavioral factors affecting parental hesitancy toward child seasonal influenza vaccination.

Factor	N (%)^1^
**Parental opinion on safety of vaccines**	
Strongly agree	6,379 (62.0)
Somewhat agree	2,961 (29.4)
Don't know	166 (1.8)
Somewhat disagree	418 (4.3)
Strongly disagree	255 (2.5)
**Parental opinion on effectiveness of vaccines**	
Strongly agree	5,875 (56.5)
Somewhat agree	3,551 (35.7)
Don't know	166 (1.7)
Somewhat disagree	404 (4.2)
Strongly disagree	197 (1.9)
**Parental opinion on safety of flu vaccines**	
Strongly agree	5,882 (56.6)
Somewhat agree	2,903 (28.8)
Don't know	595 (6.2)
Somewhat disagree	536 (5.6)
Strongly disagree	267 (2.8)
**Parental opinion on effectiveness of flu vaccines**	
Strongly agree	3,654 (34.4)
Somewhat agree	3,809 (37.0)
Don't know	687 (7.1)
Somewhat disagree	1,496 (15.8)
Strongly disagree	545 (5.7)
**Responding parent hesitancy**^2^ **in vaccinating child against flu**	
Hesitant	2,236 (23.7)
Not hesitant	7,514 (76.3)
**Impact of COVID-19 pandemic on parental decision to vaccinate their children against flu**	
More likely	1059 (10.6)
Less likely	954 (9.7)
No impact	7976 (79.7)
**Reasons why parents were hesitant to vaccinate their children against the flu** ^3^	
My child is not at risk of getting the flu or at risk of severe infection	950 (42.8)
I wanted to first discuss the flu vaccine with my child’s health care practitioner	94 (3.9)
I was concerned about the effectiveness of the flu vaccine	773 (35.0)
I had concerns about the safety of the flu vaccine and/or side effects	660 (29.4)
My child had a bad experience with previous vaccines	141 (6.2)
I did not know where to get reliable information	54 (2.1)
Religious or philosophical reasons	107 (4.8)
My child had a bad experience with healthcare providers	27 (1.1)
Concerns about racism or discrimination	15 (0.7)
Never get flu vaccine/not necessary	101 (4.3)
Concerns about combining the COVID-19 & flu vaccines /COVID-19 vaccine was enough	69 (2.9)
Other	268 (11.4)
**Obstacles that prevented parents from vaccinating their children against the flu** ^3^	
No obstacles	1,571 (80.9)
Difficulty to book time off work/school for a vaccine appointment	431 (34.1)
Living in a remote area (limited transportation)	26 (2.0)
No reliable internet access	#
Cost of the vaccine	32 (2.7)
Language barrier	8 (0.5)
Concerns about racism or discrimination	11 (0.9)
My child fears needles	320 (24.3)
Other	578 (44.1)

# High sampling variability or small sample size.

1 Percent weighted by region, child’s age group, and child’s sex at birth.

2 Vaccine hesitancy refers to a delay in acceptance or refusal of vaccines despite availability.

3 Multiple response options could be selected by respondents.

### Determinants of non-vaccination

**Figure 2:**
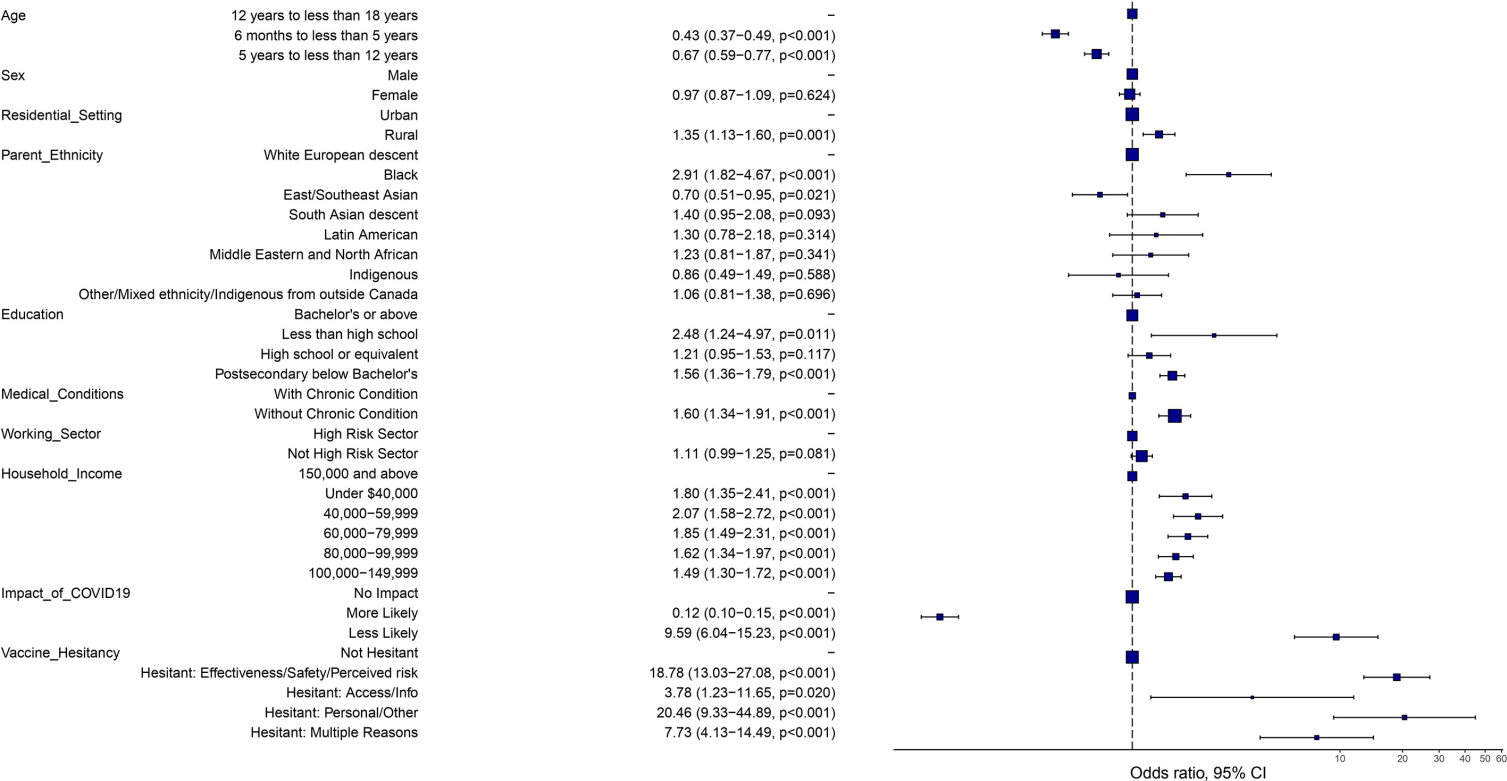
Determinants of seasonal influenza non-vaccination in Canadian children aged 6 months to 17 years (expressed as odds ratios based on multivariable logistic regression).

As detailed in Table 4, the multivariable logistic regression models incorporated variables that were identified as predictors for non-vaccination against seasonal influenza in children. Independent predictors of non-vaccination included the child being in the older age group (12–17 years) compared to younger children (less than 12 years of age), residence in rural locales rather than urban settings (aOR: 1·35, 95% CI: 1·13–1·60, *p* < 0·001), and parental educational attainment below the level of a Bachelor’s degree compared to those with a Bachelor’s degree or above. Ethnicity of the parent was also found to be a predictor of non-vaccination, but its impact was not uniform across all groups, with children of Black parents significantly more likely to be non-vaccinated compared to children of White European descent (aOR: 2·91, 95% CI: 1·82, 4·67, *p* < 0·001). Children in lower household income tiers were associated with a significantly higher odds of non-vaccination compared to the highest income bracket (>$150,000): under $40,000 (aOR: 1·80, 95% CI: 1·35–2·41, *p* < 0·001) and $40,000–$59,999 (aOR: 2·07, 95% CI: 1·58–2·72, p < 0·001). Additionally, parents who perceived the COVID-19 pandemic as a deterrent to seasonal influenza vaccination were considerably more likely to refrain from vaccinating their child (aOR: 9·59, 95% CI: 6·04, 15·23, p < 0·001). Reasons given by respondents for their reluctance to vaccinate their children were strong predictors of non-vaccination. For example, parental hesitancy due to concerns about seasonal influenza vaccine effectiveness, safety, and perceived risk of infection were associated with a significant high odds of non-vaccination compared to non-hesitant parents (aOR: 18·78, 95% CI: 13·03, 27·08, *p* < 0·001). Figure 2 provides a visual representation of the multivariable logistic regression model’s findings, which display the aORs and their 95% confidence intervals for each predictor of seasonal influenza non-vaccine.

**Table 4:** Multivariable logistic regression analysis: identifying key predictors of seasonal influenza non-vaccination in children.

Characteristic	Adjusted Odds Ratio		
	aOR^1^	95% CI^2^	*p*-value
**Child age**			<0·001
12 years to less than 18 years	—	—	
6 months to less than 5 years	0·43	0·37, 0·49	<0·001
5 years to less than 12 years	0·67	0·59, 0·77	<0·001
**Child Sex**			0·624
Male	—	—	
Female	0·97	0·87, 1·09	0·624
**Urban/Rural setting**			<0·001
Urban	—	—	
Rural	1·35	1·13, 1·60	<0·001
**Ethnicity of responding parent**			<0·001
White European descent	—	—	
Black	2·91	1·82, 4·67	<0·001
East/Southeast Asian	0·70	0·51, 0·95	0·021
South Asian descent	1·40	0·95, 2·08	0·093
Latin American	1·30	0·78, 2·18	0·314
Middle Eastern and North African	1·23	0·81, 1·87	0·341
Indigenous	0·86	0·49, 1·49	0·588
Other/mixed ethnicity/Indigenous from outside Canada	1·06	0·81, 1·38	0·696
**Education of responding parent**			<0·001
Bachelor’s or above	—	—	
Less than high school	2·48	1·24, 4·97	0·011
High school or equivalent	1·21	0·95, 1·53	0·117
Postsecondary below Bachelor’s	1·56	1·36, 1·79	<0·001
**Existing medical condition of child**			<0·001
With chronic condition	—	—	
Without chronic condition	1·60	1·34, 1·91	<0·001
**Working sector of responding parent**			0·081
High risk sector	—	—	
Not high risk Sector	1·11	0·99, 1·25	0·081
**Total household income ($)**			<0·001
150,000 and above	—	—	
Under 40,000	1·80	1·35, 2·41	<0·001
40,000–59,999	2·07	1·58, 2·72	<0·001
60,000–79,999	1·85	1·49, 2·31	<0·001
80,000–99,999	1·62	1·34, 1·97	<0·001
100,000–149,999	1·49	1·30, 1·72	<0·001
**Impact of COVID-19 pandemic on parental decision to vaccinate children against influenza**			<0·001
No impact	—	—	
More likely	0·12	0·10, 0·15	<0·001
Less likely	9·59	6·04, 15·23	<0·001
**Reasons for parental hesitancy to vaccinate child against influenza**			<0·001
Not hesitant	—	—	
Hesitant: effectiveness/safety/perceived risk	18·78	13·03, 27·08	<0·001
Hesitant: access/info	3·78	1·23, 11·65	0·020
Hesitant: personal/other	20·46	9·33, 44·89	<0·001
Hesitant: multiple reasons	7·73	4·13, 14·49	<0·001

1 aOR = adjusted odds ratio.

2 CI = confidence interval.

## Discussion

Using data from a nationally representative sample of Canadian parents/guardians with children under 18 years of age, this study explores factors influencing parental decisions to vaccinate children against seasonal influenza. Notably, during the 2021–2022 influenza season, a substantial majority of 70% of the children within this age bracket remained unvaccinated. The survey data indicated that prior to the COVID-19 pandemic, only 24% of children received the influenza vaccine every influenza season, with 43% of parents surveyed reporting their child had never received the vaccine. Interestingly, in light of the potential impact of the pandemic on Canadians’ view on vaccination, 80% of parents or guardians indicated that the pandemic did not alter seasonal influenza vaccination decisions for their children.

The great majority (81%) of parents or guardians whose children remained unvaccinated reported encountering no barriers to the seasonal influenza vaccination process. At the same time, about a quarter (24%) of parents or guardians expressed hesitancy toward the vaccination of their children against seasonal influenza. This hesitancy stemmed predominantly from a perceived lack of risk associated with the disease and reservations about the vaccine’s efficacy and safety, suggesting that both the threat from seasonal influenza infection and the advantages of vaccination are underestimated. The diminished perception of risk correlates directly with reduced vaccine uptake, a pattern consistent with findings from broader international research (8, 16).

In the present study, sociodemographic factors were strongly correlated with vaccination decisions. Specifically, our analysis demonstrated a decreasing likelihood of non-vaccination with decreasing age, a finding that aligns with previous research exploring determinants of seasonal influenza vaccination in children prior to the onset of the COVID-19 pandemic (17, 18). This pattern is particularly important given that younger children with seasonal influenza often manifest symptomatic infections at higher rates, require antibiotic treatments, and on occasions experience severe complications (19, 20).

While there is some evidence that sex may play a role in seasonal influenza vaccine coverage among children (21–23), we did not find significant differences in seasonal influenza vaccine uptake between male and female children. However, disparities emerged when examining the influence of parental ethnicity on vaccination patterns. Notably, children of Black parents exhibited an almost three-fold increase in the likelihood of non-vaccination relative to those of White European descent. Such disparities highlight the possibility of varied barriers to vaccination across different ethnic groups, a theme supported by prior studies (24–26).

Geographic differences also had a substantial influence on vaccination coverage. Children residing in urban settings typically exhibit a higher likelihood of receiving the seasonal influenza vaccine, which may be largely attributed to greater accessibility to health services (27). In the present study, children from rural areas were 35% more likely to be unvaccinated compared to their urban peers. This trend corroborates earlier findings of lower seasonal influenza vaccination coverage observed among children living in rural settings, irrespective of factors like the child’s age, maternal educational background, family income, or the number of children in the same household (27). Educational attainment of parents emerged as an important determinant of vaccination choices. Previous research suggests that parents with lower educational attainment generally have reduced literacy rates, potentially affecting their understanding of the risks associated with seasonal influenza on their children (28–31). Similarly, our analysis revealed that parents with lower levels of education are more likely to have non-vaccinated children compared to those with a university degree. Additionally, the COVID-19 pandemic emerged as a determinant of parental decisions on vaccinating their children: although the majority of respondents indicated that the pandemic did not influence their decision regarding seasonal influenza vaccination for their child, those who felt deterred by the pandemic exhibited markedly increased odds of having their child unvaccinated. This observation aligns with recent research, which identified changes in risk perception due to COVID-19 as a significant factor influencing caregivers’ intentions to vaccinate their children against seasonal influenza (32).

The findings from this analysis underscore the complexity and multifaceted nature of parental hesitancy toward vaccinating their children against seasonal influenza. Concerns related to seasonal influenza vaccine effectiveness, safety, and perception of seriousness of the seasonal influenza infection emerged as a strong predictor of child non-vaccination. Despite the availability of various resources in Canada to inform families about the importance of influenza vaccination in children, such as the Canadian Immunization Guide by the Public Health Agency of Canada (33), influenza prevention infographics and factsheets by Immunize Canada (34), and detailed efficacy data from the NACI in its Canadian Immunization Guide Chapter on Influenza and Statement on Seasonal Influenza Vaccine (35), parental concerns about the effectiveness of the vaccine in children persist. This observation aligns with other studies, which have also highlighted that parents’ comprehension of influenza’s severity and the potential safety and adverse effects of the vaccine play a pivotal role in the seasonal influenza vaccine uptake and decisions to vaccinate their children (28, 36–38). Such findings resonate with earlier studies that spotlight the influence of prior vaccination behaviors on current decisions (39), weaving a narrative of the complex interplay between historical experiences and present-day vaccination decisions (40–42).

It is evident that many parents’ decisions to vaccinate or not are anchored in their perceptions of risk, often shaped by incorrect or incomplete information (43, 44), including concerns that the vaccine might induce influenza or unfounded fears about potential side effects (45). In this study, parents expressing hesitancy due to concerns about the vaccine’s effectiveness, safety, or their perception of risk had significantly higher odds of not vaccinating their children than parents who were not hesitant. Likewise, parents who cited multiple reasons for their hesitancy, access or information-related hesitancy concerns, and those who expressed personal or other reasons for hesitancy were also strongly associated with non-vaccination. This underscores the need of accurate information dissemination, clear public health messages about the potential seriousness of influenza infection in children, and addressing parental concerns about the safety and efficacy of the seasonal influenza vaccine to enhance immunization rates among children. In light of these challenges, short-term interventions can play a pivotal role in improving vaccine coverage. Implementing face-to-face communication interventions allows for direct engagement with parents, effectively conveying the importance of influenza vaccination. Furthermore, expanding digital platforms can facilitate the dissemination of accessible, science-backed information, empowering parents to make informed decisions. Additionally, launching targeted educational campaigns directed at parents and caregivers can highlight the benefits of influenza vaccination, emphasizing its role in preventing severe illness, reducing hospitalizations, and safeguarding vulnerable populations. By employing these strategies, we can enhance immunization rates among children, contributing to the broader goal of public health protection.

Strengths of our study include the large sample size; a detailed examination of sociodemographic factors, parental and child vaccine uptake history; and questions on parental knowledge, attitudes, and beliefs, including the impact of COVID-19 pandemic. To our knowledge, this study is the first to assess children’s seasonal influenza vaccination in Canada during the COVID-19 pandemic, and utilizing a nationally representative sample. We also acknowledge specific limitations of the study. As protocol called for surveying just one adult (parent/guardian) and one child from each family, the vaccination status of other siblings within the family was not examined. With an overall response rate of 26.1%, there remains the possibility of non-response bias. However, different strategies were employed to minimize the potential risk of this bias, including utilizing local phone number outpulsing and offering the survey in Canada’s two official languages. Although reliance on self-reported data introduces the possibility of recall bias, the likelihood of such bias is presumably low since respondents were reflecting on vaccinations within the last six months.

## Conclusion

This survey underscores the complexity of parental decisions regarding vaccinating their children against seasonal influenza. Key determinants influencing non-vaccination decisions included lower household income, lower parental educational attainment, the influence of the COVID-19 pandemic as a deterrent, and various facets of vaccine hesitancy. These findings offer valuable insights for policymakers and public health professionals, emphasizing the need for tailored strategies to overcome barriers, especially in lower-income families. By addressing parental concerns, including safety apprehensions and misconceptions about seasonal influenza vaccination, and implementing informed, targeted public health initiatives, these strategies can effectively alleviate hesitancy and enhance vaccination uptake, thereby contributing to better overall public health outcomes.

## Data availability statement

The data analyzed in this study is subject to the following licenses/restrictions: Detailed results, tables, and the methodological report can be accessed through the Library and Archives Canada website. Survey results can be accessed at: https://www.canada.ca/en/public-health/services/immunization-vaccines/vaccination-coverage/childhood-covid-19-immunization-coverage-survey-2022-results.html. Requests to access the dataset should be directed to: ccics-ecvec@phac-aspc.gc.ca.

## Ethics statement

The studies involving humans were approved by the Health Canada and the Public Health Agency of Canada Research Ethics Board. Participants provided their informed consent to participate in the survey. The survey was also approved by the Privacy Management Division of the Public Health Agency of Canada. The studies were conducted in accordance with the local legislation and institutional requirements. The participants provided their written informed consent to participate in this study.

## Author contributions

AA: Conceptualization, Formal analysis, Methodology, Visualization, Writing – original draft, Writing – review & editing. SD: Conceptualization, Validation, Writing – review & editing. CU: Conceptualization, Validation, Writing – review & editing. ME: Conceptualization, Supervision, Writing – review & editing. DK: Conceptualization, Methodology, Supervision, Writing – review & editing. JL: Conceptualization, Supervision, Writing – review & editing.

## Footnotes

^1^Key sub-populations for quotas included: parents with children aged 0–4, 5–11, and 12–17 years; parents from all provinces and territories; and a gender balance among children (50% males, 50% females).

^2^ *As participants could select multiple options, percentages do not add to 100%.*
