# Supplementary material for: Determinants of influenza non-vaccination among Canadian children: insights from a nationwide survey
Source: Front Public Health. 2024 Jun 5;12:1400782. doi: 10.3389/fpubh.2024.1400782 (PMC11188407; doi:10.3389/fpubh.2024.1400782)

**Determinants of Influenza Non-Vaccination Among Canadian Children: Insights from a Nationwide Survey**

**Supplementary Material**

**Figure S1. Patterns of Missing Data Across Variables in the Dataset**

This figure presents graphical summaries that visualize the patterns of missingness across variables with over 5% missing data, facilitating an in-depth assessment of the randomness of these missing values.


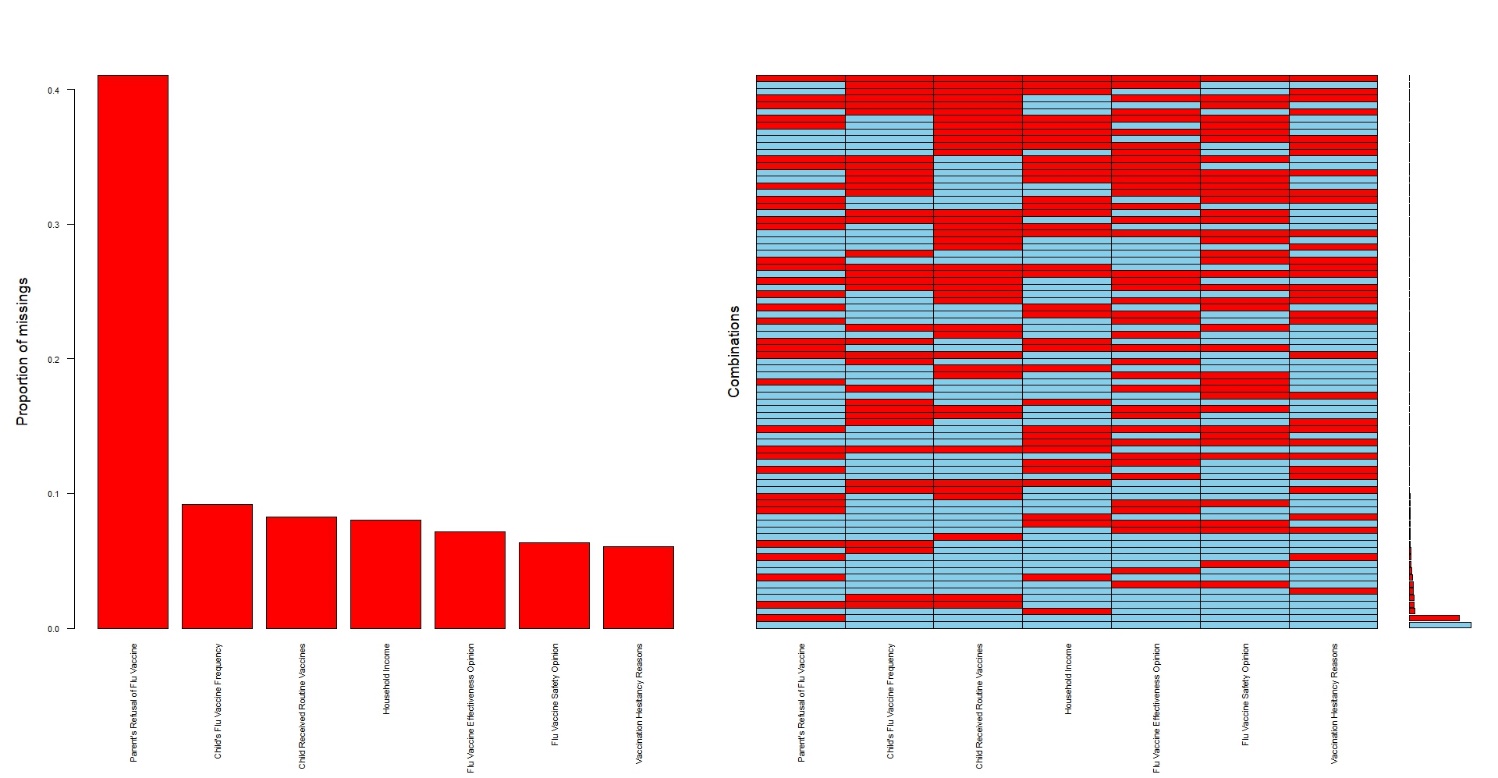

Supplement: Supplementary file 1 [file Data_Sheet_1.docx]
